# PATTERNS OF SOCIAL CARE USE WITHIN THE OLDER POPULATION: WHAT CAN WE LEARN FROM ROUTINELY COLLECTED DATA?

**DOI:** 10.1093/geroni/igad104.2294

**Published:** 2023-12-21

**Authors:** Kelly Brotherhood, Barbara Hanratty, Gemma Spiers, Camila Caiado, Julia Newton

**Affiliations:** Newcastle University, Newcastle upon Tyne, England, United Kingdom; Newcastle University, Newcastle upon Tyne, England, United Kingdom; Newcastle University, Newcastle upon Tyne, England, United Kingdom; Durham University, Durham, England, United Kingdom; Newcastle University, Newcastle upon Tyne, England, United Kingdom

## Abstract

Research with routinely collected social care data has untapped potential to inform new care delivery approaches and techniques. To identify opportunities for service improvement and enhance our understanding of care pathways experienced by the older population, we collaborated with a local authority in the North East of England. We set out to characterise the use of social care services and associated outcomes within the local older population (aged 65+). 171,386 records were extracted from the local authority’s social care case management system, relating to 38,191 unique individuals across the last 40 years. We identified the care packages provided to the local population, including care provided in care homes (with and without nursing), private households and assisted living facilities. The study population varied in terms of the number of care packages provided to each individual (median 7 packages, IQR 4-11) and the average duration of individual care packages (median 41 days, IQR 14 - 274 days). The care pathways that are most common amongst the older population will be described, including sequencing and outcomes, and grouped by the reason for providing care (e.g., respite, long-term care) and the reason why each care package ended (e.g., death, returning home). The wide range of care pathways experienced demonstrate the heterogeneity in needs and preferences within the older population. This dataset and analyses are an invaluable way of identifying areas of potential unmet need and evaluating the effectiveness of short-term care services.

